# Analysis of binary traits: testing association in the presence of linkage

**DOI:** 10.1186/1471-2156-6-S1-S92

**Published:** 2005-12-30

**Authors:** Gudrun Jonasdottir, Juni Palmgren, Keith Humphreys

**Affiliations:** 1 Department of Mathematical Statistics, Stockholm University, Sweden; 2 Department of Medical Epidemiology and Biostatistics, Karolinska Institute, Sweden

## Abstract

Most methods for testing association in the presence of linkage, using family-based studies, have been developed for continuous traits. FBAT (family-based association tests) is one of few methods appropriate for discrete outcomes. In this article we describe a new test of association in the presence of linkage for binary traits. We use a gamma random effects model in which association and linkage are modelled as fixed effects and random effects, respectively. We have compared the gamma random effects model to an FBAT and a generalized estimating equation-based alternative, using two regions in the Genetic Analysis Workshop 14 simulated data. One of these regions contained haplotypes associated with disease, and the other did not.

## Background

Testing association in a region with confirmed linkage may increase the rate of false positives in family-based studies. In a linked region one expects similarity between related individuals. If unaccounted for, this similarity may be mistaken for association. Different remedies have been suggested, ranging from using a robust variance estimator [1] for the general test statistic FBAT (family-based association tests) [2] to a model-based approach in which the linkage is modelled in the covariance structure [3] (VCM, variance components model). The VCM has been developed for continuous traits, while FBAT can be used with both binary and continuous traits. In this article we concentrate on methods for testing association in the presence of linkage, using binary traits. We compare the program FBAT for binary traits to both the gamma random effects (GRE) method and also a GEE (generalized estimating equation) [4] approach. For the purpose of our comparisons we have used the simulated Genetic Analysis Workshop 14 (GAW14) data. We have compared the three methods' ability to pick up a signal in a region with association, as well as their ability to avoid signalling in a region with no association.

## Methods

We consider a random effects model for binary events, which is similar in spirit to the multivariate survival model in Zhong and Li [5], which models association and linkage as fixed effects and random effects, respectively. We use a result for random effects models for binary outcomes, which has been described by Conaway [6]. It is shown that for gamma distributed random effects, the unconditional distribution of the outcome using a log-log link can be written as a sum of easily calculated terms. Analytical tractability is only achievable for a few other combinations of random effects distributions and link functions, such as the beta distribution with a log(-log) link [6]. The random effects model in Zhong and Li [5] assigns one random effect for each of the two alleles of the father and one random effect for each of the two alleles of the mother. The notion of inheritance vector is used to describe the alleles for all family members jointly. The method presented here works for all sizes of sibships, and may also be easily adapted to extended pedigrees.

### GRE model

Let (*Y*_*i*1_, *Y*_*i*2_, ..., ) be the binary trait vector for family *i *and let *j *denote offspring (*j *= 1, 2, ..., *J*_*i*_). We allow for different family sizes *J*_*i*_. We use *θ*_*mj *_and *θ*_*pj *_to denote the effect of the transmitted alleles to offspring *j*, with *m*_*j *_= 1, 2 the maternal alleles and *p*_*j *_= 3, 4 the paternal alleles, respectively. Conditional on the transmitted alleles, we write the probability of the trait for offspring *j *in family *i *as *P*(*Y*_*ij *_= 1|*θ*_*mj*_, *θ*_*pj*_). We consider a model with a log(-log) link of the form

log(-log(*P*(*Y*_*ij *_= 1|*θ*_*mj*_, *θ*_*pj*_))) = log(*θ*_*mj *_+ *θ*_*pj*_) + ***X***_***j***_***β***,

or equivalently



The effects *θ *of the transmitted alleles act multiplicatively on the offspring trait probability, and the effect of each transmitted allele is multiplied by a term involving the parameter vector ***β ***describing the fixed genetic effects. Following Li [7] and Li and Zhong [8] we assume that the maternal and paternal alleles are independent and that each allele contributes an effect to the trait which is random and follows a gamma distribution with scale *α*/2 and shape *λ*. The model has a tractable closed form for the joint unconditional trait probabilities for the offsprings in a sibship. Let Ψ denote all ordered subsets of 1, 2, ..., *J*_*i*_, Ψ = {{0}, {1}, {2}, {1, 2}, {3}, ..., {1, 2, ..., *J*_*i*_}. Let  denote the joint unconditional probability of *Y*_*ij *_= 1 for all j ∈ *T*, where *T *∈ Ψ. Calculating the probability  requires integrating over *θ*_1_, *θ*_2_, *θ*_3 _and *θ*_4_. There is a tractable solution [6]. It turns out that



The elements of vector ***a***_*k*_, *a*_*jk*_, indicate whether allele *k *has been transmitted to offspring *j*, *j *= 1, 2, ..., *J*_*i*_. The probabilities for all *T *∈ Ψ can be placed in a vector π*. It has been shown [6] that the unconditional probability for all possible outcomes of ***Y ***can be written as ***π *= *Z***^**-1**^π*.

The matrix ***Z ***indicates all subsets of *T*. In order to get the probability of the observed *Y*_*ij *_one needs only to pick the corresponding row in *π.*. In Table 1, an example of *T*, matrix ***Z ***and vector ***π ***for three sibs is given. The likelihood for the observed data, for families *i *(*i *= 1, 2, ..., *n*), is

.

We used the statistical software **R** (version 1.9.1) [9] to implement the likelihood and maximize it with respect to the association parameter ***β***.

We have so far not described how to deal with incompletely observed inheritance vectors. In the context of testing association in the presence of linkage, Zhong and Li [5] suggest using GENEHUNTER to obtain the distribution for inheritance vectors at any arbitrary point along the chromosome. In our single-point analysis we treat all inheritance vectors compatible with the data as equally likely and construct a weighted mean of *π*_i_. We return to the choice of weights in the discussion.

### FBAT and GEE

We compare the GRE with FBAT (version 1.5.1) [2] and a generalized estimating equation (GEE)-based alternative [4]. For FBAT we assume a linear allele-dose model, and for the GEE-based alternative we assume a linear allele-dose on the logit scale and an exchangeable covariance structure.

We used FBAT option *-o *to find the optimal weight. We then applied the optimal weight to the phenotype score and used FBAT option *-e *to test our data. The function *gee *(in package *gee*) in **R** (version 1.9.1) was used for the GEE analysis. The *gee *package can be found at the **R** web page [9].

### GAW14 simulated data

For details concerning how the simulation was performed see the GAW14 Data Description [10].

All analyses were performed with knowledge of the data simulation process. We chose to analyze the data with respect to trait A. Trait A is known to be associated with haplotypes in the Region D3, while markers in the D2 region are known to not be associated with trait A. For the purpose of our comparison we therefore chose to "purchase" markers in the D3 region (B05T4135–B05T4142) as well as markers from the D2 region (B03T3048–B03T3067). Our aim was to use regions D2 and D3 to gain some insight into the performance of the different methods. More specifically, we were not expecting a signal in region D2, but were hoping for one in region D3.

The Aipotu population (one of four simulated populations) only consists of nuclear families, although these are of different sizes. For simplicity, we chose to concentrate on the Aipotu population and to only include families of maximum size six (i.e., two parents and at most four offspring).

We merged 10 (out of 100) replicates in order to get a sample with reasonable power. This provided us with a total of 481 independent nuclear families. There was no missing data and we did not simulate any.

We selected the markers described above and analyzed each marker separately in a set of single-point analyses. The method we have described can, however, be extended to multiple markers and a multipoint analysis.

## Results

**Figure 1:**
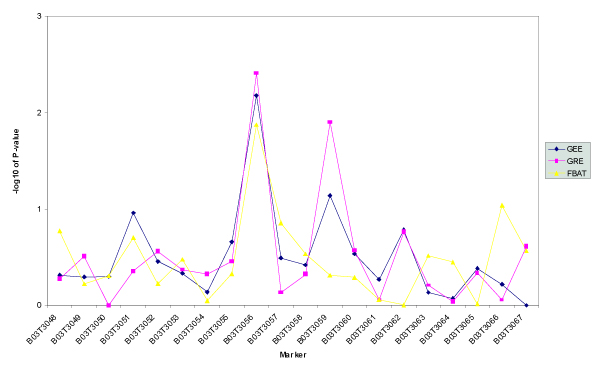
Trait A region D2, -log10 of the *p*-values.

**Figure 2:**
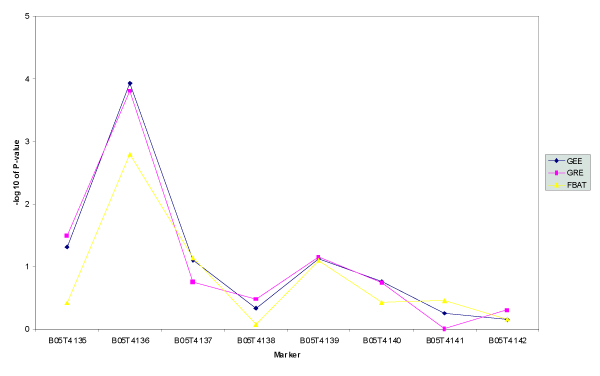
Trait A region D3, -log10 of the *p*-values.

We analyzed the ten merged replicates in regions D2 and D3 and we were able to identify interesting markers in both regions. In region D2, all three methods (FBAT, GEE and GRE) indicated marker B03T3056 had borderline significance with a *p*-value of around 0.01 (Figure 1). The peak was slightly less using FBAT. In Region D3, which harbored a haplotype-based association in the simulated data, we were able to detect association with marker B05T4136. The detected association had a slightly smaller *p*-value when GEE and GRE (*p*-value ~0.0001) were used, compared with the FBAT procedure (Figure 2).

## Conclusion

In the simulated data, region D2 harbored no locus associated with trait A. All three methods (FBAT, GEE, and GEE) gave a signal for association with marker B03T3056 with a *p*-value around 0.01. However, taking the multiple testing into account, this *p*-value does not reach statistical significance. The results from all markers in the region are showed in Figure 1. Across the markers, no one method produced consistently higher or lower *p*-values than any other method.

In region D3, association with trait A was simulated at the haplotype level. We still chose to perform single-point analyses with each marker in turn. The GEE and the GRE turn out to be slightly better in detecting significant markers than FBAT.

The GRE model presented here seems to work well, compared to both GEE and FBAT. It would be useful to perform simulation studies to assess validity and power of the three procedures under different genetic models. The GRE model requires more computational time, stemming from the fact that in spite of the closed form in (3) it is time consuming to evaluate and to maximize the likelihood.

A problem with the GRE model is how to handle the missing information on transmission. In our single-point algorithm we propose using a weighted sum (with equal weights) over all compatible inheritance vectors, given parental and offspring genotypes. Following Zhong and Li [5] we compute the distribution over inheritance vectors without attention to phenotype. However, given that linkage is assumed, the probabilities of transmission are not invariant to offspring phenotypes. It would be useful to investigate the impact of using our suboptimal weights on the GAW data, and more generally in comparing the validity and power of the different approaches using simulations under different genetic models.

## Abbreviations

FBAT: Family based association tests

GAW14: Genetic Analysis Workshop 14

GEE: Generalized estimating equation

GRE: Gammar random effects

VCM: Variance components model

## Figures and Tables

**Table 1:** Matrices for *J*_*i *_= 3 offspring

*T*	*Z *matrix^b^								*π *subscripts^c^		
		
ø	1	1	1	1	1	1	1	1	1	1	1
1	1	0	1	0	1	0	1	0	0	1	1
2	1	1	0	0	1	1	0	0	1	0	1
1, 2	1	0	0	0	1	0	0	0	0	0	1
3	1	1	1	1	0	0	0	0	1	1	0
1, 3	1	0	0	0	0	0	0	0	0	1	0
2, 3	1	1	0	0	0	0	0	0	1	0	0
1, 2, 3	1	0	0	0	0	0	0	0	0	0	0

^a^All ordered combinations of 1, 2, 3. Each row in *T *corresponds in choosing a set off offspring (*j *= 1, 2, 3) and setting their trait.

*Y*_*ij *_= 1.  is the probability of offspring set *T *being affected.

^b^The matrix for which π* = *Zπ*. The inverse of *Z *is used to calculate *π*, given π*.

^c^The subscripts for matrix *π*. For example, row two corresponds to the outcome *Y*_*i*1 _= 0, *Y*_*i*1 _= 1, and *Y*_*i*3 _= 1.
